# Amorphous silica nanoparticles size-dependently aggravate atopic dermatitis-like skin lesions following an intradermal injection

**DOI:** 10.1186/1743-8977-9-3

**Published:** 2012-02-02

**Authors:** Toshiro Hirai, Tomoaki Yoshikawa, Hiromi Nabeshi, Tokuyuki Yoshida, Saeko Tochigi, Ko-ichi Ichihashi, Miyuki Uji, Takanori Akase, Kazuya Nagano, Yasuhiro Abe, Haruhiko Kamada, Norio Itoh, Shin-ichi Tsunoda, Yasuo Yoshioka, Yasuo Tsutsumi

**Affiliations:** 1 Laboratory of Toxicology and Safety Science, Graduate School of Pharmaceutical Sciences, Osaka University, 1-6, Yamadaoka, Suita, Osaka 565-0871, Japan; 2 Laboratory of Biopharmaceutical Research (Pharmaceutical Proteomics), National Institute of Biomedical Innovation, 7-6-8, Saito-Asagi, Ibaraki, Osaka 567-0085, Japan; 3 The Center for Advanced Medical Engineering and Informatics, Osaka University, 1-6, Yamadaoka, Suita, Osaka 565-0871, Japan

**Keywords:** Nanoparticle, Silica, Allergy, Cytokines

## Abstract

**Background:**

Due to the rising use of nanomaterials (NMs), there is concern that NMs induce undesirable biological effects because of their unique physicochemical properties. Recently, we reported that amorphous silica nanoparticles (nSPs), which are one of the most widely used NMs, can penetrate the skin barrier and induce various biological effects, including an immune-modulating effect. Thus, it should be clarified whether nSPs can be a risk factor for the aggravation of skin immune diseases. Thus, in this study, we investigated the relationship between the size of SPs and adjuvant activity using a model for atopic dermatitis.

**Results:**

We investigated the effects of nSPs on the AD induced by intradermaly injected-mite antigen *Dermatophagoides pteronyssinus *(Dp) in NC/Nga mice. Ear thickness measurements and histopathological analysis revealed that a combined injection of amorphous silica particles (SPs) and Dp induced aggravation of AD in an SP size-dependent manner compared to that of Dp alone. In particular, aggravation was observed remarkably in nSP-injected groups. Furthermore, these effects were correlated with the excessive induction of total IgE and a stronger systemic Th2 response. We demonstrated that these results are associated with the induction of IL-18 and thymic stromal lymphopoietin (TSLP) in the skin lesions.

**Conclusions:**

A particle size reduction in silica particles enhanced IL-18 and TSLP production, which leads to systemic Th2 response and aggravation of AD-like skin lesions as induced by Dp antigen treatment. We believe that appropriate regulation of nanoparticle physicochemical properties, including sizes, is a critical determinant for the design of safer forms of NMs.

## Background

With the development of nanotechnology, practical uses for nanomaterials (NMs) are rapidly spreading to a wide variety of fields, such as cosmetics, food, and medicine, because they have unique physicochemical properties and exert innovative functions [1-3]. These observations mean that intentional exposure of NMs is unavoidable in everyday life. However, it is a concern that NMs can exhibit unknown harmful effects [4,5]. For example, maternal exposure to titanium dioxide nanoparticles (nTiO_2_) induces gene expression alterations related to brain development [6]. We also revealed that amorphous silica nanoparticles (nSPs) and nTiO_2 _induce reproductive and/or liver toxicity compared with submicron-sized amorphous silica particles (SPs) [7,8]. However, little information exists on the potential hazard of NMs. To ensure the safety of NMs and enjoy their many benefits, it is essential to obtain more information on the relationship between the factors associated with NM hazards and their physicochemical properties, such as size and surface properties, to design safer forms of NMs.

nSPs are used as a base material in cosmetics and an anti-caking agent in food because of their high transparency and coatability [9,10]. Because nSPs are one of the most widely used NMs, the chances of being exposed to nSPs in our daily life are high. Thus, we must analyze the *in vitro*/*in vivo *biodistribution of nSPs including estimations of whether they can penetrate the biological barrier. Additionally, we need to estimate whether they could be responsible for acute/chronic side effects, thereby facilitating a more accurate risk analysis of nSPs. Under these circumstances, we revealed that nSPs with a diameter of 70 nm (nSP70) can penetrate the skin barrier and enter keratinocytes and Langerhans cells [11]. Our studies further revealed that a dermal application of nSP70 induces apoptosis in dermal cells [11]. In addition, we reported that nSPs have a greater cytotoxic effect on Langerhans cells [12]. Thus it is possible that nSPs may be associated with development of the allergic diseases such as atopic dermatitis (AD). However, the relationship between the size of SPs and its adjuvant activity which can affect aggravation of AD like skin lesion has remained unclear.

In present study, we investigated the effect of nSPs on AD-like skin lesions using *Dermatophagoides pteronyssinus *(Dp), which is the main allergen of AD [13], in NC/Nga mice. We also examined the relationship between the SP size and aggravation of AD.

## Results

### Physicochemical properties of variously sized SPs

Prior to undertaking this study, we first analyzed the physicochemical properties of SPs (Table 1). SPs remained as stable well-dispersed particles in PBS and not as aggregates. Thus, these particles were ideally suited to evaluate whether their biological effects depended on particle size.

**Table 1:** Summary of the physicochemical properties of amorphous silica particles (SPs).

	Primary particle **size (nm)**^**a**^	Diameter in PBS (nm)	Mean zeta potential (mV)	pH
mSP1000	1000	1136 ± 32.1	-33.2 ± 1.4	7.6
nSP300	300	264 ± 7.2	-25.8 ± 0.7	7.4
nSP100	100	106 ± 0.6	-24.3 ± 0.5	7.4
nSP70	70	76 ± 1.7	-19.5 ± 1.0	7.4
nSP30	30	39 ± 4.2	-14.0 ± 1.3	7.4

Mean particle size and zeta potential in solution of silica particles are expressed as mean ± S.D. (n = 3). ^a ^Information from technical datasheet of products.

### Effects of variously sized SPs on AD-like skin lesions

**Figure 1:**
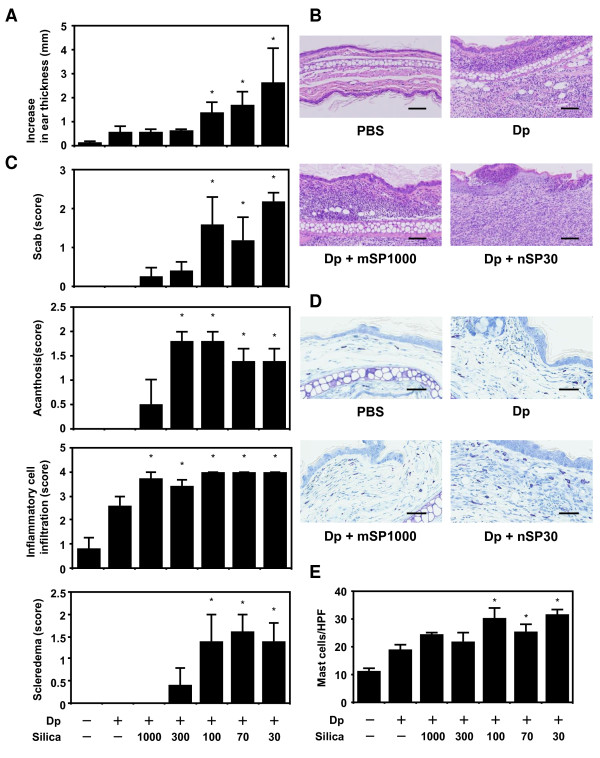
**Effects of amorphous silica nanoparticles (nSPs) on atopic dermatitis (AD)-like skin lesions induced by *Dermatophagoides pteronyssinus *(Dp)**. NC/Nga mice were intradermally injected on the ventral side of both ears with Dp alone or Dp plus SPs in PBS on days 1, 3, 5, 7, 9, 11, 13, 16, and 18. Ear thickness was measured on days 0 and 19 (A). The ears were removed 24 h after the last intradermal injection. Sections were prepared, and the ears were stained with H&E to assess representative symptoms of AD (B and C) or with toluidine blue to assess mast cell infiltration (D and E). Representative histological findings of the PBS, Dp, Dp + mSP1000, and Dp + nSP30 groups are shown. Several representative symptoms of AD were scored in the H&E sections (C). Infiltration of mast cells was evaluated as the number of cells in toluidine blue sections in a high power field (E). Data are presented as mean ± SE of 4-5 animals per group. The results are representative of two separate experiments. *P < 0.05 vs. Dp group. Scale bars: 100 μm (B) and 50 μm (D).

To evaluate whether variously sized SPs affects AD-like skin lesions induced by Dp, we first measured ear thickness on days 0 and 19. We confirmed that the intradermal injection of Dp enhanced ear thickening as compared to that of PBS (Figure 1A). A combined injection of Dp and the submicron-sized SPs, mSP1000 or nSP300, did not enhance ear thickening compared with that of Dp alone. In contrast, a combined injection of Dp + nSPs (nSP100, nSP70, and nSP30) enhanced ear thickening two- to fivefold compared with that of Dp alone. nSP-treatment showed a marked tendency to cause ear thickening. Thus, nSPs with a diameter ≤100 nm acted specifically to enhance ear thickening in this model.

Next, we performed hematoxylin-eosin and toluidine blue staining using ear sections 24 h after the last intradermal injection (Figure 1B and 1D). Then, several representative symptoms (scab, acanthosis, inflammatory cell infiltration, and scleredema) were scored on the H&E sections (Figure 1C). A Dp injection caused significant inflammatory cell infiltration compared with a PBS injection. In contrast, a combined injection of Dp and variously sized SPs caused more severe inflammatory cell infiltration than that of Dp alone. Although the Dp injection did not cause scabs, acanthosis, or scleredema, the combined injection of Dp and SPs with a diameter ≤300 nm caused acanthosis. Furthermore, a combined injection of Dp and nSPs caused significant scabs and scleredema (Figure 1C). Mast cell infiltrates in the skin were stronger following the combined injection of Dp and nSPs (Figure 1D and 1E). Thus, the injection of Dp and submicron-sized SPs caused only inflammatory cell infiltration and acanthosis compared with that of Dp alone. However, injecting Dp and nSPs caused not only inflammatory cell infiltration and acanthosis but also scabs and scleredema. These findings suggest that injecting nSPs with a diameter ≤100 nm and Dp induced severe AD-like histological changes.

### Effects of variously sized SPs on the IgE response

**Figure 2:**
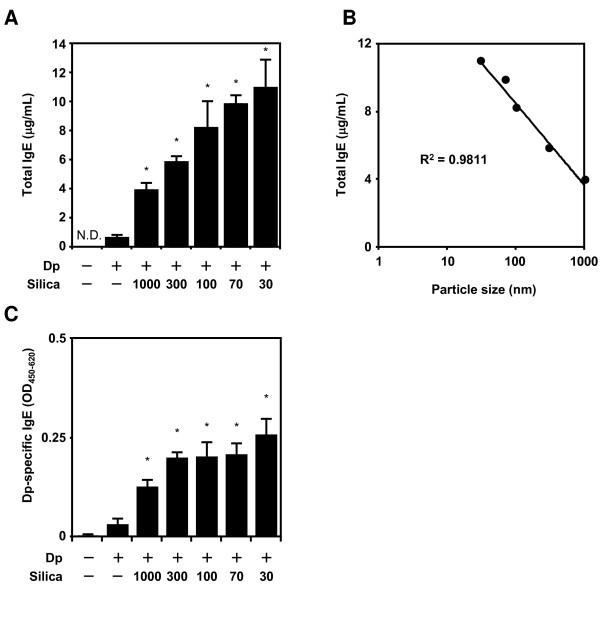
**Effects of amorphous silica particles (SPs) on total and *Dermatophagoides pteronyssinus *(Dp)-specific IgE production in plasma**. NC/Nga mice were intradermally injected on the ventral side of both ears with Dp alone or Dp plus SPs in PBS on days 1, 3, 5, 7, 9, 11, 13, 16, and 18. To evaluate AD severity, plasma was collected 24 h after the last intradermal injection and analyzed by ELISA for total and Dp-specific IgE (A and C). A regression analysis of total IgE was also performed (B). Data are presented as mean ± SE of 4-5 animals per group. The results are representative of two separate experiments. *P < 0.05 vs. Dp group.

Many reports have shown that the total IgE and allergen-specific IgE level correlate with severity of AD [14,15]. To clarify the nSP-mediated aggravation of AD-like lesions, we first measured total IgE and Dp-specific IgE 24 h after the last intradermal injection (Figure 2). The total IgE in the Dp alone group was higher than that in the PBS group (Figure 2A). Furthermore, the levels of total IgE in the Dp + SP groups were enhanced in an SP size-dependent manner compared with those in the Dp alone group. A regression analysis showed that the particle size reduction was a significant contributor to this effect (Figure 2B; R^2 ^= 0.98). Therefore, it paralleled the severity of the AD-like skin lesions. In the presence of variously sized SPs, the Dp-specific IgE levels were significantly increased compared with Dp alone group (Figure 2C). However, Dp-specific IgE levels were not paralleled with the size of SPs. Thus, nSP-mediated aggravation of AD-like lesions has more to do with total IgE than Dp-specific IgE.

### Cytokine expression analysis of nSP-injected skins

**Figure 3:**
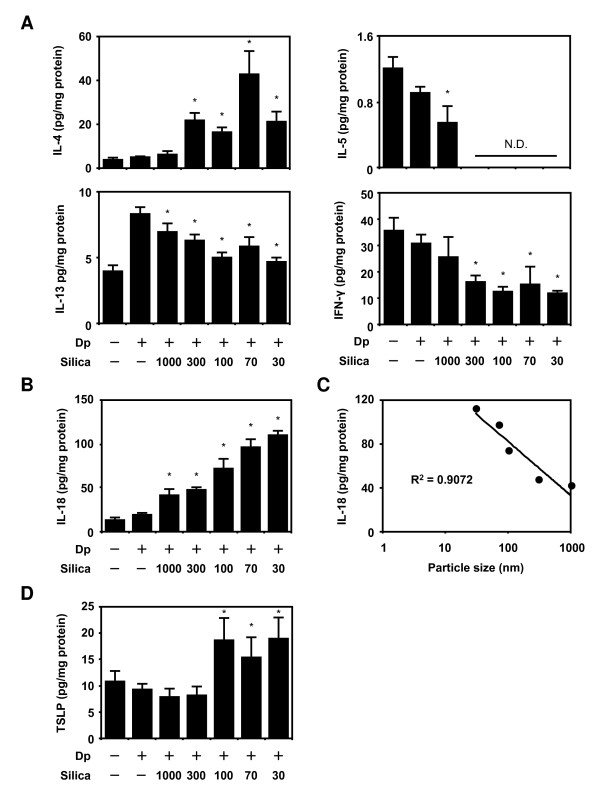
**Analysis of the local immune response induced by *Dermatophagoides pteronyssinus *(Dp) + amorphous silica particles (SPs)**. NC/Nga mice were intradermally injected on the ventral side of both ears with Dp alone or Dp plus SPs in PBS on days 1, 3, 5, 7, 9, 11, 13, 16, and 18. At 24 h after the last intradermal injection, the ears were removed and homogenized. The homogenates were centrifuged at 10,000 rpm for 5 min, and the supernatants were collected. The levels of interleukin (IL)-4, IL-5, IL-13, interferon (IFN)-γ, IL-18, and thymic stromal lymphopoietin (TSLP) in the supernatants were examined by ELISA (A, B, and D). A regression analysis of IL-18 was also performed (C). Data are presented as mean ± SE of 4-5 animals per group. The results are representative of two separate experiments. *P < 0.05 vs. Dp group.

To clarify the mechanism of AD-like skin lesions enhanced by SPs, we analyzed the cytokine expression profile in the skin related to Dp. We examined the levels of Th2-type cytokines (IL-4, IL-5, and IL-13), Th1-type cytokines IFN-γ, IL-18, and thymic stromal lymphopoietin (TSLP) in the ears 24 h after the last intradermal injection (Figure 3). Intradermal injection of Dp alone increased the local expression of IL-4 and IL-13 and decreased the production of IL-5 and IFN-γ compared with PBS injection. Only differences of the expression of IL-13 between the Dp group and PBS group is significant. In contrast, the level of IL-4 in the Dp + SP groups with diameters ≤300 nm increased significantly compared with that in the Dp alone group. The levels of IL-5 and IL-13 in the Dp + SP groups with a diameter ≤300 nm decreased significantly compared with those in the Dp alone group. Additionally, injections of SPs with a diameter ≤300 nm led to a significant decrease in IFN-γ compared to those of Dp alone (Figure 3A). Thus, the injection of SPs with Dp tended to enhance the effects of injecting Dp alone in terms of cytokine levels in the skins. However, the levels of IFN-gammma and IL-5 were significantly reduced after SPs treatment when compared to Dp only. The levels of IL-18, known as an AD-inducing cytokine, were higher in the Dp + smaller SP groups than those in the Dp alone group (Figure 3B). A regression analysis showed that the particle size reduction was a significant contributor to this effect (Figure 3C; R^2 ^= 0.91). IL-18 production size-dependently increased similar to that of plasma total IgE levels. Furthermore, the levels of TSLP, an AD-inducing cytokine, were enhanced in only the Dp + nSP groups compared with those in the Dp alone group (Figure 3D). This result matched that of increase in ear thickness.

### Dp-specific immune response in Dp + SPs-injected mice

**Figure 4:**
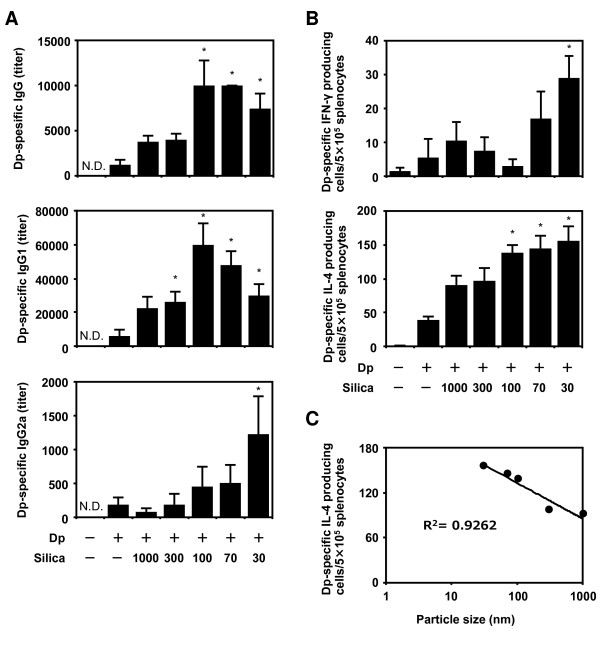
**Analysis of the systematic immune response induced by *Dermatophagoides pteronyssinus *(Dp) + amorphous silica particles (SPs)**. NC/Nga mice were intradermally injected on the ventral side of both ears with Dp alone or Dp plus SPs in PBS on days 1, 3, 5, 7, 9, 11, 13, 16, and 18. At 24 h after the last intradermal injection, splenocytes and plasma were collected from each group. Dp-specific IgG and its subclasses (A) in plasma were analyzed by ELISA. Splenocytes from each group were cultured with 100 μg ml^-1 ^Dp. The levels of Dp-specific interferon (IFN)- γ and interleukin (IL)-4-producing cells were examined by individual cytokine-specific ELISPOT assay (B). A regression analysis of Dp-specific IL-4-producing cells was also performed (C). Data are presented as mean ± SE of 4-5 animals per group. The results are representative of two separate experiments. *P < 0.05 vs. Dp group.

To evaluate the effect of SPs on systemic immune responses, we examined the levels of Dp-specific IgG and its subtypes by ELISA 24 h after the last intradermal injection (Figure 4A and 4B). Higher levels of Dp-specific IgG were observed in the Dp + SP groups than those in the Dp alone group (Figure 4A). Furthermore, higher Dp-specific IgG levels were observed, particularly in Dp + nSP groups. Dp-specific IgG1 levels in the Dp + SP groups were higher than those in the Dp alone groups. In contrast, the levels of Dp-specific IgG2a were low in all groups except the Dp + nSP30 group. Plasma IgG subclass responses have been used to assess the type of immune response. It is well-known that IgG1 is indicative of a Th2-type response, whereas IgG2a is indicative of a Th1 response. Therefore, these results indicate that Dp + SPs induced antigen-specific Th2-type immune responses and that Dp + nSP30 induced not only Th2-type immune responses but also Th1-type responses.

To further characterize the type of systemic immune response, we measured the levels of Dp-specific IFN-γ- and IL-4-secreting splenocytes from each intradermally injected mouse using a cytokine-specific ELISPOT assay (Figure 4B). The number of Dp-specific IFN-γ-secreting splenocytes in the Dp + nSP30 group was significantly greater than that in all other groups. However, no difference was observed in the Dp-specific IFN-γ-secreting splenocytes of all groups except the Dp + nSP30 group. These levels of Dp-specific IFN-γ-secreting splenocytes were correlated with Dp-specific IgG2a levels. The levels of Dp-specific IL-4-secreting splenocytes in the Dp + SP groups were enhanced in a SP size-dependent manner compared with those in the Dp alone group (Figure 4C). The regression analysis showed that the particle size reduction was a significant contributor to this effect (Figure 4D; R^2 ^= 0.93). Thus, the levels of Dp-specific IL-4-secreting splenocytes paralleled AD severity.

## Discussion

The lifetime prevalence of AD is estimated at 15-30% in children and 2-10% in adults, and the incidence of AD has increased by two- to threefold during the past three decades in industrialized countries [16,17]. Although environmental factors and lifestyle changes are speculated to be associated with the increased number of patients with AD, the detailed relationships between environmental factors or lifestyle changes and the increase in AD are not well understood. Some epidemiological studies have shown that chronic exposure to urban fine particles could be a risk factor for AD [18,19]. In addition, nTiO_2 _and polystyrene nanoparticles have been reported to exacerbate AD [20,21]. Thus, among other environmental factors, NMs may be a risk factor for the onset or aggravation of AD. However, the relationship between AD and nSPs, which are one of the most widely used NMs in various applications, has never been investigated. Here, to clarify the relationship between NM use and the onset or aggravation of AD, we investigated the effects of nSPs on AD-like skin lesions induced by a mite allergen Dp.

Typical symptoms of AD, such as inflammation or itching of the skin, are induced by chemical mediators such as histamine and some cytokines [22,23]. Furthermore, these chemical mediators or cytokines are induced by IgE or antigen cross-linking of IgE bound to FcεRI on mast cells [24]. We measured Dp-specific IgE to determine allergen-specific IgE participation in the aggravation of AD. As expected, the Dp-specific IgE level was higher in the Dp + SP groups than that in the Dp alone group (Figure 2C). Because particle materials influence the intracellular processing of antigen in antigen-presenting cells (APCs) such as dendritic cells (DCs) [25,26], it is possible that SPs affected these APCs and changed the Dp-specific immune responses. However, these Dp-specific IgE levels were not dependent on SP size and were not high in the Dp + nSP groups compared with those in the Dp + submicron-sized SPs groups. In contrast, a regression analysis showed that the total IgE levels, which are known to correlate with AD severity [27,28], were completely dependent on injected SP size (Figure 2A; R^2 ^= 0.98). Thus, this result suggested that smaller SPs induced more severe AD and, most importantly, that the mechanisms of AD aggravation caused by SPs were induced not only by Dp-specific IgE. Thus, the mechanisms different from the mechanisms correlated with Dp-specific IgE production were also involved in this model. Therefore, clarification of the mechanisms of size-dependent aggravation of AD-like skin lesions is important for a risk analysis of nSPs and for designing safer forms of nSPs.

AD skin lesions are characterized by recruitment of lymphocytes, monocytes, eosinophils, and mast cells [29,30]. These infiltrating cells produce Th2 cytokines including IL-4 and IL-13. These mediators promote Th2 deviation not only in acute eczematous skin lesions but also in systemic adaptive immune systems [31,32]. This Th2 deviation could further compromise barrier function in the skin lesions and complete a potential outside-inside-outside pathogenic loop of AD [33]. Additionally, IL-18 or TSLP plays a critical role in the spontaneous development of AD [34,35]. Considering this, we first performed an analysis of the Th1/Th2 cytokine balance in the skin lesions to clarify the mechanism of AD aggravation by SPs. Although IL-4 levels in the ears increased, IL-5, IL-13, and IFN-γ levels decreased following exposure to Dp + SPs compared with those following exposure to Dp alone (Figure 3). There is possible that variously sized SPs induced the expression of Th2 cytokines at an early phase and that this induction might contribute to the aggravation of symptoms. A time-course study is needed to elucidate the correlation between symptoms and inflammatory mediators.

Epithelial cells are major cell source of various pro-allergic cytokines, such as IL-18 and TSLP. As they are accumulated preferentially in the inflammatory sites of patients with AD, IL-18 and TSLP might be involved in the development of AD [16,36]. To reveal the mechnisms of the nSPs-mediated aggravation of AD-like skin lesion, we performed further analyses to measure IL-18 and TSLP levels in the skin. Interestingly, the IL-18 levels in skin lesions of the Dp + SP groups increased compared to those in the Dp alone group in a SP size-dependent manner (Figure 3; R^2 ^= 0.91). IL-18 is a cytokine that stimulates mast cells and basophils to release AD-associated molecules, Th2 cytokines, and histamine, without a pathway that involves Ag, IgE, or FcRI. Furthermore, IL-18 induces large amounts of total IgE via natural killer T and CD4^+ ^T cells [37-39]. These effects of IL-18 finally induce AD-like skin lesions [34]. Considering these, this IL-18 production in a size-dependent manner of SPs show why smaller SPs induced severe AD accompany the production of total IgE. On the other hands, the TSLP levels were only increased following the exposure to Dp + "nSPs" (Figure 3). This result is similar to those of increase in ear thickness, scab and scleredema. TSLP can promote the Th2 cytokine-associated inflammation by directly promoting the effector functions of CD4^+ ^Th2 cells, basophils and other granulocyte populations via DC [35,40]. Furthermore, it is reported that skin-specific overexpression of TSLP resulted in an AD-like skin lesion [35,40]. Thus, these results suggested that TSLP as well as IL-18 might be involved in the development of nSP-mediated AD-like skin lesions in mice. Although we need further mechanistic studies for understanding the nSP-mediated production of IL-18 and TSLP, modification of physicochemical nSPs properties for suppressing IL-18 or TSLP production might be value in the development of safer NMs.

On the other hands, we observed that only treated with nSP70 did not change pathology and local immune responses compared with PBS treated mouse except IL-18 productions (our preliminary data). However only IL-18 production was tendency to be enhanced by treatment with only silica nanoparticles, the enhancement of IL-18 productions are very slightly (not significant). Thus, we think the evoked inflammation in the ear was mainly attributed to the mixture of nSPs and Dp. Therefore we also must give attention to the interactions of nSPs with antigen to develop of safer NMs.

### Conclusions

In this study, we showed the intradermal injections of SPs and Dp antigen aggravate AD-like skin lesions in size dependent manner. We speculated that particle size affects SPs-induced IL-18 and TSLP inductions enhance the Th2 immune responses related to Dp and then induce aggravation of AD-like skin lesions. On the other hands, the further studies are required to analyze the mechanisms why IL-18 and TSLP are induced by nSPs. Furthermore, the studies are also required to investigate whether SPs are the risk factor of AD using topical applications of SPs, which is a more likely mode of exposure to NMs. Considering the fact that the nSPs already used in general are smaller than our used ones, our results demand a careful hand. We consider that appropriate regulation of physical and chemical properties of nanoparticles, including sizes mediated by the inductions of IL-18 and TSLP are critical determinants for the design of safer forms of NMs.

## Methods

### SPs

Suspensions of SPs (Micromod Partikeltechnologie GmbH, Rostock, Germany) (25 and 50 mg ml^-1 ^in water) were used; particle size diameters were 1000, 300, 100, 70, and 30 nm (designated as mSP1000, nSP300, nSP100, nSP70, and nSP30, respectively). These SPs don't have any coating. SP suspensions were stored at room temperature. The suspensions were sonicated in power of 400 W for 5 min at 25°C and then vortexed for 1 min immediately prior to use.

### Mice

Male NC/Nga slc mice were purchased from Nippon SLC (Kyoto, Japan) and used at 8 weeks of age. We used n = 4 or 5 animals per each assay. All animal experimental procedures were performed in accordance with the institutional ethical guidelines for animal experiments.

### Physicochemical examination of SPs

SPs were diluted to 0.25 mg ml^-1 ^(nSP30 and nSP70) or 0.5 mg ml^-1 ^(nSP100, nSP300, and mSP1000) with PBS (Sigma-Aldrich, St. Louis, MO, USA), and the average particle size and zeta potential were measured using a Zetasizer Nano-ZS (Malvern Instruments Ltd., Worcestershire, UK) at 25°C. The mean size and size distribution of SPs were measured using the dynamic light scattering method. The zeta potential was measured by laser Doppler electrophoresis. We perform these measurements using Size and Zeta capillary cell (Malvern Instruments Ltd). The pH of each particle suspension was measured with an ISFET pH meter (Shindengen, Tokyo, Japan).

### Study protocols

Mice were divided into seven experimental groups (4 or 5 mice were used in each group): PBS, Dp, Dp + mSP1000, Dp + nSP300, Dp + nSP100, Dp + nSP70, and Dp + nSP30 groups. The PBS group received 20 μl of PBS. The Dp group received 2.5 μg of Dp mite allergen extract (Cosmo Bio LSL, Tokyo, Japan) in 20 μl PBS. The Dp + mSP1000, Dp + nSP300, Dp + nSP100, Dp + nSP70, and Dp + nSP30 groups received a combined administration of 2.5 μg Dp and 250 μg SPs (1000, 300, 100, 70, or 30 nm) in 20 μl PBS. Mice were intradermally injected on the ventral side of both their ears with Dp alone or Dp plus SPs in PBS on days 1, 3, 5, 7, 9, 11, 13, 16, and 18 under anesthesia with pentobarbital (somnopentyl; Kyouritsuseiyaku, Tokyo, Japan). The amount of injected liquid was 10 μl per ear. We measured ear thickness on days 0 and 19.

### Histological analysis

At 24 h after the last intradermal injection, the ears of mice were removed and placed in fixative solution (4% paraformaldehyde in PBS; Wako, Osaka, Japan). A histopathological examination was performed by the Applied Medical Research Laboratory (AMRL; Osaka, Japan). Several representative symptoms (scab, acanthosis, inflammatory cell infiltration, and scleredema) observed in the AD of each sample were scored as 0 (none), 1 (very mild), 2 (mild), 3 (moderate), or 4 (severe). Scoring was also performed by AMRL, who was unaware of the treatment assignments. Mast cell numbers in three high-power fields at ×400 magnification were selected randomly and counted.

### Blood sampling

Mice were anesthetized with pentobarbital 24 h after the last intradermal injection. The chest and abdominal walls were opened, and blood was sampled by cardiac puncture. Then blood was centrifuged at 3000 g at 4°C and we obtained plasma. Plasma was stored at -80°C until assay.

### Quantitation of total IgE Abs

Total IgE in plasma was measured with an ELISA assay kit (BD Biosciences, San Diego, CA, USA) according to the manufacturer's instructions.

### Detection of Dp-specific Ab by ELISA

Antigen-specific Ab levels in plasma were determined by ELISA. ELISA plates (Maxisorp, type 96F; Nunc A/S, Roskilde, Denmark) were coated with 100 μg ml^-1 ^Dp in coating buffer PBS and incubated overnight at 4°C. The plates were washed with PBS-Tween20, incubated with blocking solution (Block Ace; Dainippon Sumitomo Pharmaceuticals, Osaka, Japan) at room temperature (RT) for 2 h, and plasma dilutions were added to the antigen-coated plates. After a 2-h RT incubation, the coated plates were washed with PBS-Tween20 and incubated with a HRP-conjugated goat anti-mouse IgG or IgG1 solution or a biotin-conjugated goat anti-mouse IgG2a or IgE detection Ab solution (Southern Biotechnology Associates, Birmingham, AL, USA) at RT for 2 h. To detect IgG2a or IgE, the plates were washed with PBS-Tween20 and then incubated with HRP-coupled streptavidin (Southern Biotechnology Associates) for 30 h at RT. After the incubation, the color reaction was developed with tetra methyl benzidine (MOSS, Inc.; Pasadena, MD, USA), stopped with 2 N H_2_SO_4_, and measured at OD_450-620 _on a microplate reader.

### Isolation of splenocytes

Spleens were aseptically removed and placed in RPMI 1640 (Wako) supplemented with 10% FBS, 10 ml L^-1 ^of a 100 × nonessential amino acid solution (Gibco, Invitrogen, Carlsbad, CA, USA), 50 μM 2-mercaptoethanol (Gibco), and 1% antibiotic cocktail (Pennicilin; 10000 U/mL, Streptomycin; 10000 μg/mL, amphotericin B; 25 μg/mL) (Gibco). A single-cell suspension of splenocytes was treated with ammonium chloride to lyse the red blood cells, which were washed, counted, and suspended in RPMI 1640 to a final concentration of 1 × 10^7 ^cells ml^-1^.

### ELISPOT assay

An ELISPOT assay was performed to detect interferon (IFN)- γ and IL-4 producing cells. Dp-specific cytokine responses were evaluated by culturing the splenocytes (5 × 10^5 ^cells well^-1^) stimulated with Dp (100 μg ml^-1^) *in vitro*. After a 24-h incubation at 37°C, the plate was washed, and the IFN-γ- and IL-4-producing cells were measured with an ELISPOT assay kit (BD Biosciences) according to the manufacturer's instructions.

### Quantitation of cytokine protein levels in ear tissue supernatants

At 24 h after the last intradermal injection, mice ears were removed and stored at -80°C. Later, the ears were homogenized with cell extraction buffer (Invitrogen). The homogenates were then centrifuged at 10,000 rpm for 5 min, and the supernatants were stored at -30°C. ELISAs for IFN-γ (BD Biosciences), IL-4, IL-5, IL-13 (eBioscience, San Diego, CA, USA), IL-18 (MBL, Nagoya, Japan), and thymic stromal lymphopoietin (TSLP; R&D Systems, Minneapolis, MN, USA) in the ear tissue supernatants were performed according to the manufacturer's instructions.

### Statistical analysis

All data are presented as mean ± SE. The significant differences among groups were determined by ANOVA. Differences between the experimental groups and the control group were determined by Williams' test. P < 0.05 was considered significant.

## Competing interests

The authors declare that they have no competing interests.

## Authors' contributions

TH and TY designed the study; TH, HN, TY, S. Tochigi, KI, MU and TA performed experiments; TH and TY collected and analyzed data; TH and TY wrote the manuscript; KN, YA, YY, HK, NI and S. Tsunoda gave technical support and conceptual advice. YT supervised the all of projects. All authors discussed the results and commented on the manuscript. All authors have read and approved the final manuscript.
